# Interactive Effect of Arbuscular Mycorrhizal Fungi (AMF) and Olive Solid Waste on Wheat under Arsenite Toxicity

**DOI:** 10.3390/plants12051100

**Published:** 2023-03-01

**Authors:** Mha Albqmi, Samy Selim, Mohammad M. Al-Sanea, Taghreed S. Alnusaire, Mohammed S. Almuhayawi, Soad K. Al Jaouni, Shaimaa Hussein, Mona Warrad, Mahmoud R. Sofy, Hamada AbdElgawad

**Affiliations:** 1 Department of Chemistry, College of Science and Arts, Jouf University, Al Qurayyat 77447, Saudi Arabia; 2 Olive Research Center, Jouf University, Sakaka 72341, Saudi Arabia; 3 Department of Clinical Laboratory Sciences, College of Applied Medical Sciences, Jouf University, Sakaka 72388, Saudi Arabia; 4 Department of Pharmaceutical Chemistry, College of Pharmacy, Jouf University, Sakaka 72341, Saudi Arabia; 5 Department of Biology, College of Science, Jouf University, Sakaka 72341, Saudi Arabia; 6 Department of Medical Microbiology and Parasitology, Faculty of Medicine, King Abdulaziz University Hospital, Jeddah 21589, Saudi Arabia; 7 Department of Hematology and Oncology, Yousef Abdulatif Jameel Scientific Chair of Prophetic Medicine Application, Faculty of Medicine, King Abdulaziz University, Jeddah 21589, Saudi Arabia; 8 Department of Pharmacology, College of Pharmacy, Jouf University, Sakaka 72341, Saudi Arabia; 9 Department of Clinical Laboratory Sciences, College of Applied Medical Sciences at Al-Quriat, Jouf University, Al-Qurayyat 77425, Saudi Arabia; 10 Botany and Microbiology Department, Faculty of Science, Al-Azhar University, Cairo 11884, Egypt; 11 Department of Botany and Microbiology, Faculty of Science, Beni-Suef University, Beni-Suef 62521, Egypt

**Keywords:** olive solid waste, AMF colonization, redox balance, photorespiration, anthocyanin metabolism

## Abstract

Heavy metal such as arsenite (As^III^) is a threat worldwide. Thus, to mitigate As^III^ toxicity on plants, we investigated the interactive effect of olive solid waste (OSW) and arbuscular mycorrhizal fungi (AMF) on wheat plants under As^III^ stress. To this end, wheat seeds were grown in soils treated with OSW (4% *w/w*), AMF-inoculation, and/or As^III^ treated soil (100 mg/kg soil). AMF colonization is reduced by As^III^ but to a lesser extent under As^III^ + OSW. AMF and OSW interactive effects also improved soil fertility and increased wheat plants’ growth, particularly under As^III^ stress. The interactions between OSW and AMF treatments reduced As^III^-induced H_2_O_2_ accumulation. Less H_2_O_2_ production consequently reduced As^III^-related oxidative damages i.e., lipid peroxidation (malondialdehyde, MDA) (58%), compared to As stress. This can be explained by the increase in wheat’s antioxidant defense system. OSW and AMF increased total antioxidant content, phenol, flavonoids, and α-tocopherol by approximately 34%, 63%, 118%, 232%, and 93%, respectively, compared to As stress. The combined effect also significantly induced anthocyanins accumulation. The combination of OSW+AMF improved antioxidants enzymes activity, where superoxide dismutase (SOD, catalase (CAT), peroxidase (POX), glutathione reductase (GR), and glutathione peroxidase (GPX) were increased by 98%, 121%, 105%, 129%, and 110.29%, respectively, compared to As^III^ stress. This can be explained by induced anthocyanin percussors phenylalanine, cinamic acid and naringenin, and biosynthesic enzymes (phenylalanine aminolayse (PAL) and chalcone synthase (CHS)). Overall, this study suggested the effectiveness of OSW and AMF as a promising approach to mitigate As^III^ toxicity on wheat growth, physiology, and biochemistry.

## 1. Introduction

Plant growth and productivity are adversely affected by metal and metalloid stress worldwide. Anthropogenic activity resulted in heavily-contaminated soils with metals and metalloid pollutants [1]. In agricultural areas, contaminated soil and water have become a serious environmental hazards, as the metalloid accumulates in edible plant parts and ultimately reaches humans [2]. Among the most ubiquitous environmental contaminants, arsenic (As) has been classified as a non-threshold class 1 carcinogen [3]. The concentration of As in soils varies with geographical regions, but its concentration can reach up to 70 mg/kg [4]. Consumption of As-contaminated agricultural products and use of As-contaminated water for drinking and irrigation comprise a palpable pathway of As exposure to the food chain, which contributes to the occurrence of As-related illnesses in indigenous people [5]. The amount of phyto-arsenic available in the soil depends upon its total and available phosphorus (P) content. As it enters the root epidermis through inorganic phosphate (Pi) channels, having a chemical similarity to P [6,7,8]. Moreover, sequestration of As in the vacuole is considered as an important strategy for As detoxification in plants [9]. The major inorganic forms of arsenic include the trivalent arsenite (As^III^ ) and the pentavalent arsenate (As^V^). Unfortunately, As is rapidly reduced to a more phytotoxic form called As^III^ that could interact with protein sulfhydryl groups and cause catastrophic cell injuries [10]. For instance, As^V^ is converted to As^III^ after it is absorbed as As^V^, producing reactive oxygen species (ROS) [11]. Plant development is negatively affected, biochemical and metabolic pathways are impeded, plant water status is disrupted, cellular integrity is broken, and nutrient absorption is restricted [11,12,13,14]. Arsinate replaces the necessary ions from adenosine triphosphate (ATP) that harm proteins, lipids, and nucleic acids, and obstructs numerous metabolic pathways, either as rival inhibitors of Pi or by interfering with the functions of enzymes, ultimately impacting development processes [15].

Plants are exposed to a number of environmental stresses, including abiotic stresses, which result in changes in structure and function that will affect growth patterns and resource allocation [16]. Plants have developed sophisticated tolerance levels, including anatomical and morphological changes to survive under As stress [16]. It also alters physiological mechanisms and metabolic performances including modification of photosynthetic machines and overproduction of osmoprotectants [13], as well as antioxidant defense biosynthesis [16]. In contrast, oxidative and ROS stress is controversial, and plants have to reduce stress-induced high ROS levels [17]. In order to enhance heavy metal tolerance mechanisms, several strategies have been suggested so far. However, environmentally-friendly approaches have received less attention [17].

Global olive oil output is rising by 5% yearly. It is predicted to yield 30 million m^3^ of OSW annually. The entire area planted with olives in the globe is around 11 million hectares, the majority of which are in the Mediterranean region [18]. Thus, the Mediterranean region’s olive oil output is steadily increasing. The solid waste created by the olive oil manufacturing process is known as olive solid waste (OSW). Managing and reusing OSW generated during the olive oil manufacturing process is a serious concern in olive oil-producing countries. The biological oxygen demand of 40–80 g/L, the chemical oxygen demand of 50–150 g/L, and the phenolic compound concentration (1739–2704 mg L^−1^) of OSW provide additional challenges for reuse and treatment [19]. Therefore, using OSW may increase soil quality indicators (aggregate stability, pH, organic matter) and plant growth performance (Mahmoud et al., 2010) because of its high mineral content, like nitrogen (N), sulfur (S), and carbon (C). It is also utilized as a soil amendment to offer energy, soil health advantages, and sequestration of metals to soil [20]. Through improved N fixation, OSW supplementation to soil enhances the availability of essential cations and mineral nutrients, including total N and P, improving water and soil fertility quality. It also helps to reduce environmental contamination and adds to higher yield and long-term production [21]. OSW has the potential to boost plant growth and production through direct or indirect methods. It improves plant development directly by boosting nutrient absorption and indirectly by improving soil physicochemical and biological properties [22].

Beneficial rhizosphere bacteria have been proposed as effective and ecologically acceptable methods of improving plant development and soil health under abiotic and biotic stress [23]. A variety of microorganisms that promote plant growth, including endophytic fungi, AMF, and plant-growth-promoting rhizobacteria, have been demonstrated to increase plant growth during stress by assessing soil fertility and structure and providing nutrients to the host plant [24]. Furthermore, AMF has been shown to increase development under harsh situations such as abiotic stress [13]. AMF is an important bio-ameliorator of stress, increasing stress tolerance and changing root architecture to enhance access to nutrients and water [25]. AMF may also boost photosynthetic activity in host plants by influencing physiological factors [25].

The second most important cereal crop worldwide is wheat (*Triticum aestivum* L.). Wheat plants may grow in a variety of climate zones (from tropical to temperate). However, several abiotic stressors, such as heavy metal toxicity impact wheat plant production and productivity. To this end, this study aimed to study As^III^ toxicity on plant growth, physiology and defense systems. Moreover, interaction with AMF application is essential for maximizing AMF’s participation in phytoremediation techniques. However, limited research has identified various harmful effects of As^III^ on plant shoot development. Arsenite a result, the present research looks at the impact of AMF inoculation on wheat growth in As^III^-contaminated soil. In the present study, treatment with OSW and AMF (*Rhizophagus irregularis*) would benefit the host plant by reducing As^III^ toxicity on growth and metabolism. However, the physiological and biochemical bases of the As^III^ stress mitigation impact of OSW and AMF are not well studied. Therefore, this study aimed to understand how the interaction between AMF and OSW can improve wheat plant growth and reduce As^III^-induced oxidative damages. To this end, photosynthesis related parameters, ascorbate–glutathione cycle, antioxidant metabolites, and enzymes and anthocyanin metabolism were investigated. Overall, this will acquire a better understanding of the underlying processes that control plant response to As^III^ toxicity.

## 2. Results

### 2.1. AMF Colonization

Heavy metal stress impacts microbial ecology, particularly in the rhizosphere. Arsenite a result, the rhizosphere microbiome is constantly changing to adapt to environmental changes. Mycorrhiza colonization in the roots and rhizosphere of wheat plants was tracked to assess AMF colonization under As^III^ stress and normal conditions (Table 1). Infection was seen in AMF-treated wheat plants, as predicted. However, compared to AMF treatment, root colonization, hyphal length, and arbuscules numbers decreased by 45, 49, and 40%, respectively, under As + AMF treatment but to a lesser extent under As + OSW+AMF treatment i.e., 40, 43, and 51%, respectively (Table 1). These results suggested that the AMF could colonize plants roots when subjected to As^III^ stress.

### 2.2. Growth Analysis

**Figure 1:**
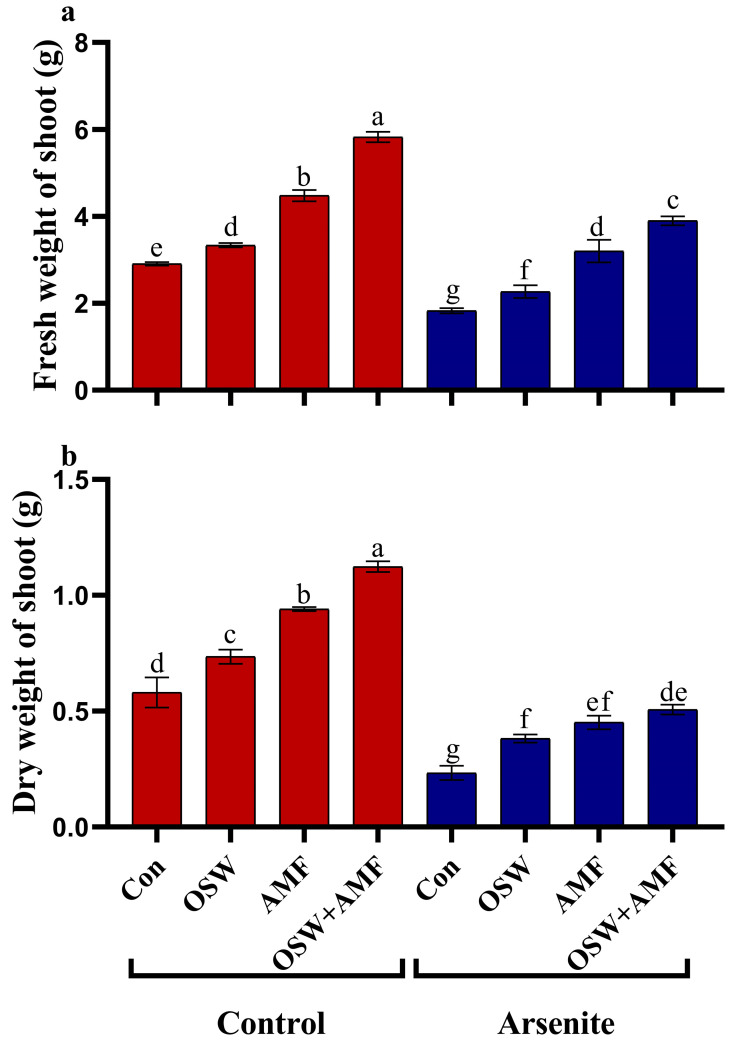
The influence of olive solid waste (OSW) and arbuscular mycorrhizal fungi (AMF), combination (OSW + AMF) on (**a**) fresh weight of shoot and (**b**) dry weight of shoots in wheat plants growing under As^III^ stress. Fishers test at *p* < 0.05 reveals significant variations in means (±standard deviation), which are different letters on the same bars.

We first tested if AMF and OSW could benefit wheat growth. We therefore quantified fresh weight (FW) and dry weight (DW) biomass production of wheat shoot. Wheat plant’s growth parameters, like fresh shoot weight and dry weight, when As^III^, OSW, AMF, and their combination (OSW + AMF) were applied (Figure 1). Applying As^III^ stress to wheat plants decreases growth parameters, such as fresh shoot weight and dry weight, which were reduced by approximately 37% and 59%, respectively, compared to non-stressed plants. OSW, AMF, and OSW + AMF, on the other hand, considerably enhanced growth parameters in As^III^ stressed plants compared to non-stressed plants. Plants treated with OSW+AMF showed the most significant increases. Consequently, the fresh weight of the shoot of plants grown in OSW+AMF-treated soil was increased by about 112% and 117% compared to the As^III^ stress.

### 2.3. Photosynthetic Rate and Pigments Contents

**Figure 2:**
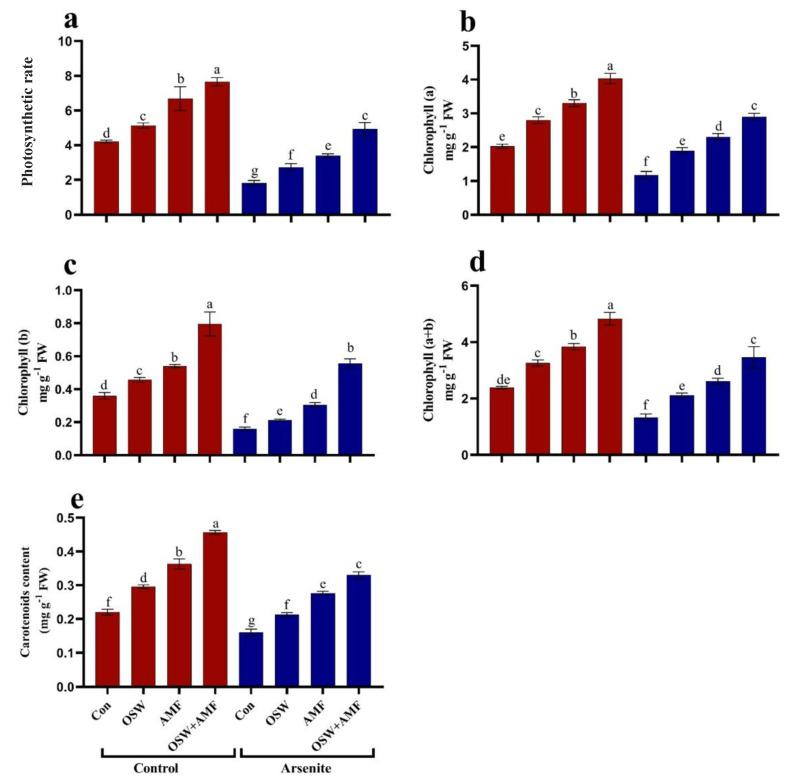
The influence of olive solid waste (OSW) and arbuscular mycorrhizal fungi (AMF), combination (OSW + AMF) on photosynthetic rate (μmol CO_2_ m^−2^ s^−1^) and photosynthetic pigments (mg/gFW) in wheat plants growing under As^III^ stress. Photosynthesis rate (**a**) Chlorophylla, (**b**) Chlorophyll b (**c**), Chlorophyll a+b (**d**), carotenoids (**e**). Fishers test at *p* < 0.05 reveals significant variations in means (±standard deviation), which are different letters on the same bars.

To investigate how AMF and OSW are able to stimulate biomass production in the presence of substantial As^III^ bioaccumulation, we quantified key parameters related to photosynthesis (total chlorophyll, carotenoid, and photosynthetic rate). Photosynthesis rate process, chlorophyll a, b, a + b, and carotenoids content in leaves of wheat plants show significant decreases of about 56%, 42.11%, 55%, 44%, and 27%, respectively, compared to control plants (Figure 2). In addition, the photosynthesis process, Chl a, Chl b, Chl a+b, and carotenoid content showed the highest values of about 169%, 147%, 247%, 159%, and 106%, respectively, after treatment with a combination of OSW+AMF compared to As^III^ stress plants followed by AMF and OSW. This significant decrease in photosynthetic activity upon As^III^ exposure could lie at the basis of reduced shoot biomass.

### 2.4. Oxidative Damage in Wheat Plants under As^III^ Stress

**Figure 3:**
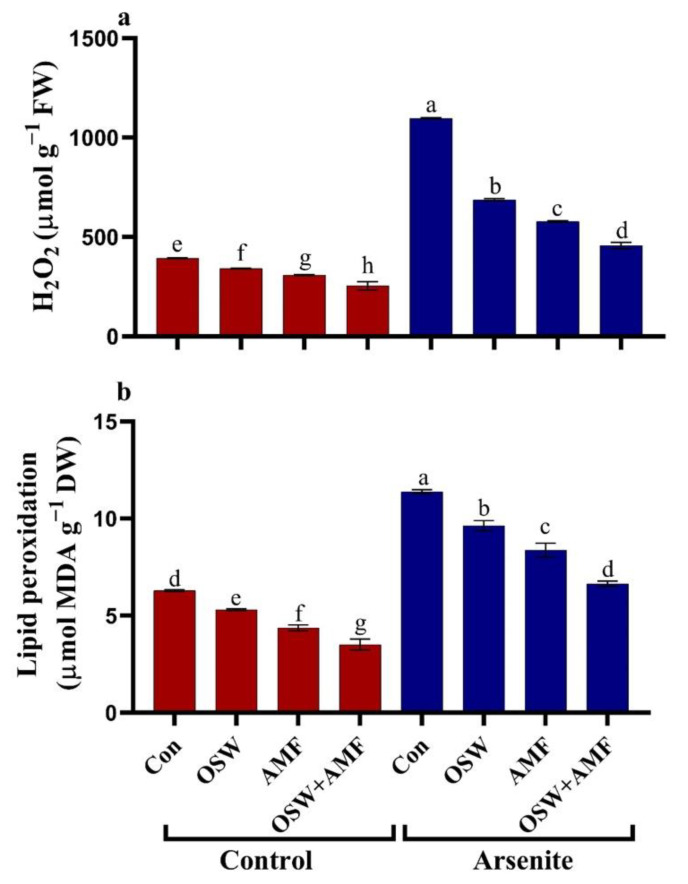
The influence of olive solid waste (OSW) and arbuscular mycorrhizal fungi (AMF), combination (OSW + AMF) on H_2_O_2_ and MDA in wheat plants growing under As stress. H_2_O_2_ level (**a**) and lipid peroxidation (MDA) (**b**). Fishers test at *p* < 0.05 reveals significant variations in means (±standard deviation), which are different letters on the same bars.

As^III^ toxicity is an important aspect of oxidative stress. Consequently, we investigated individual parameters related to oxidative damage. A significant observation on ROS generation (H_2_O_2_) and the formation of MDA included an indication of lipid peroxidation in wheat leaves treated with OSW, AMF, and a combination (OSW + AMF), in the presence or absence of As^III^ stress (Figure 3). Our findings reveal that exposing wheat plants to As^III^ stress, induced by increased As^III^, levels resulted in a considerable rise in H_2_O_2_ (179%) content compared to control plants. This led to increased lipid peroxidation (MDA 80%). On the other hand, using OSW, AMF, and a combination OSW+AMF, showed a significant capacity to ameliorate As^III^ stress by lowering the H_2_O_2_ and MDA levels. In comparison, a combination of OSW + AMF under As^III^ stress resulted in a very significant drop in H_2_O_2_ (41%) and MDA (58%) content.

### 2.5. Antioxidant Defense System

#### Antioxidant Metabolites

**Figure 4:**
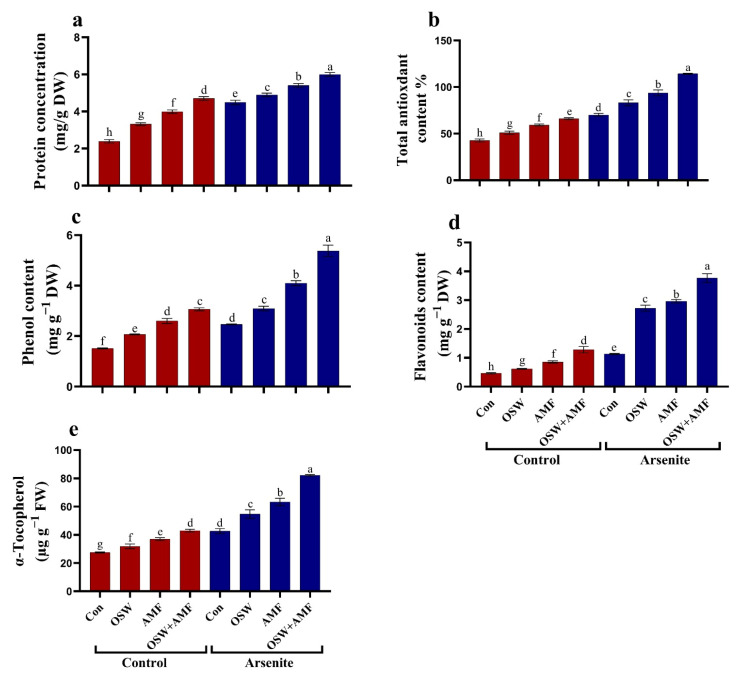
The influence of olive solid waste (OSW) and arbuscular mycorrhizal fungi (AMF), combination (OSW + AMF) on antioxidant metabolites (i.e., protein content (**a**), total antioxidant content (**b**), phenolics (**c**), flavonoids (**d**), and α-tocopherol (**e**)) in wheat plants growing under As^III^ stress. Fishers test at *p* < 0.05 reveals significant variations in means (±standard deviation), which are different letters on the same bars.

**Figure 5:**
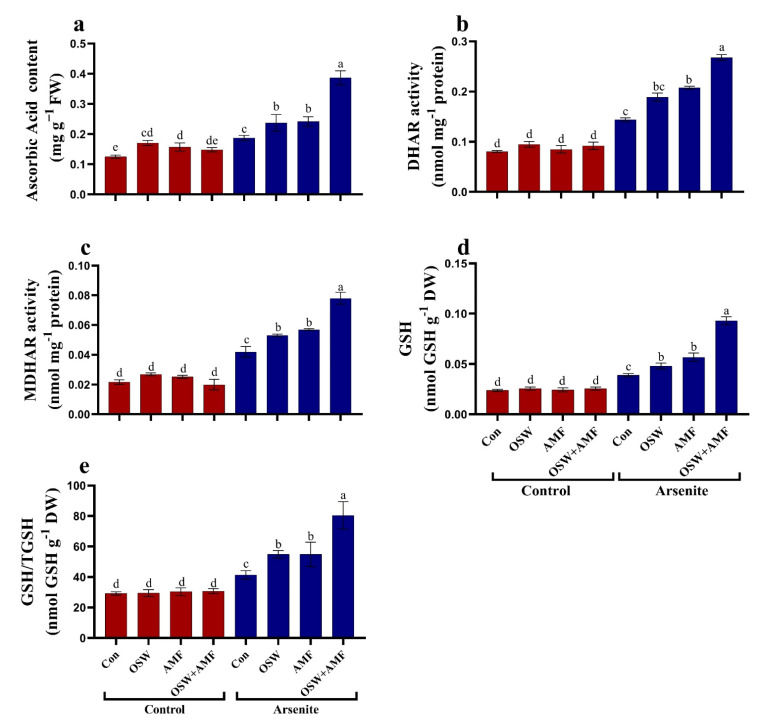
The influence of olive solid waste (OSW) and arbuscular mycorrhizal fungi (AMF), combination (OSW + AMF) on ascorbic acid (**a**), DHAR (**b**), MDHAR (**c**), GSH (**d**), and GSH/TGSH (**e**) in wheat plants growing under As^III^ stress. Fishers test at *p* < 0.05 reveals significant variations in means (±standard deviation), which are different letters on the same bars.

Next, we aimed to gain a more in-depth understanding of the biochemical processes that lie at the basis of the response to AMF and OSW under As^III^ exposure. In As^III^-stressed plants, the antioxidant metabolites (i.e., total antioxidant content, phenol, flavonoids, and α-tocopherol) increased significantly compared to the non-As plant. Treatment wheat plants with 100 mg/kg As^III^ resulted in significant increases in total antioxidant, phenol, flavonoids, and α-tocopherol content of approximately 87%, 64%, 62%, 141%, and 54%, respectively, compared to untreated plants (Figure 4). The most pronounced increases in relieving stress were observed when combination (OSW+AMF) treatment was used, followed by treatment with AMF and OSW. In As^III^ stressed wheat plants, antioxidant metabolites (i.e., total antioxidant content, phenol, flavonoids, and α-tocopherol) were significantly increased by approximately 33%, 63%, 118%, 232%, and 92%, respectively, compared to As^III^ stressed plants (Figure 4). The activities of the ASC/GSH pathway at metabolities (ASC, GSH, GSH/TGSH) and enzymes activities (DHAR, MDHAR, GSH) were markedly increased in wheat shoots under As^III^ stress compared to As^III^-stressed plants without AMF and OSW treatment (Figure 5). In addition, there was a highly significant increase in ASC levels and DHAR, MDHAR, and GSH enzymes’ activities in plants treated with combination (OSW+AMF), as compared with As stress plants (Figure 5).

### 2.6. Effects of OSW, AMF, and Combination (OSW+AMF) Application on Antioxidant Stressed Plants Were Enzymes in Wheat Plants under As^III^ Stress

**Figure 6:**
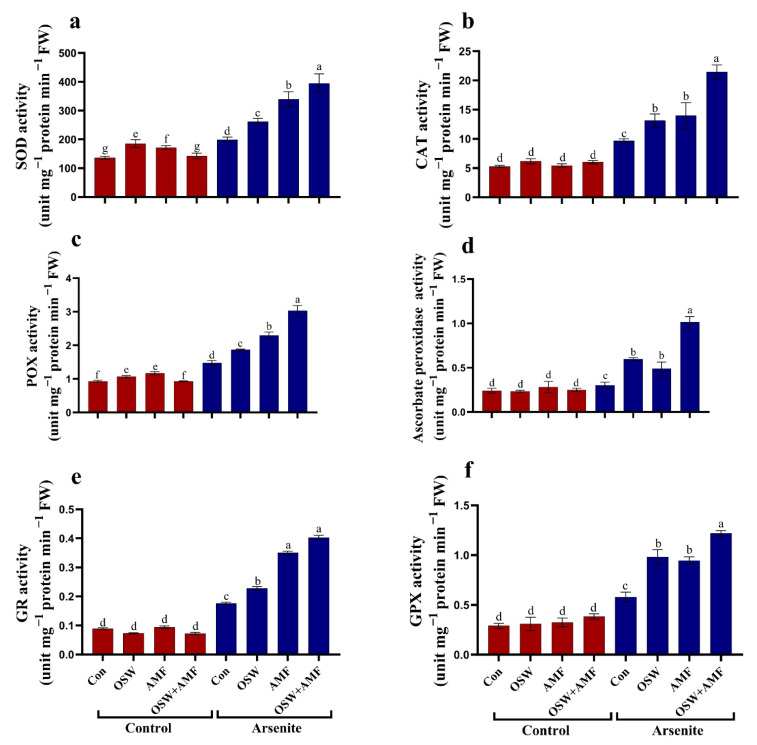
The influence of olive solid waste (OSW) and arbuscular mycorrhizal fungi (AMF), combination (OSW + AMF) on SOD (**a**), CAT (**b**), POX (**c**), ASC peroxidase (**d**), GR (**e**), and GPX (**f**) in wheat plants growing under As stress. Fishers test at *p* < 0.05 reveals significant variations in means (±standard deviation), which are different letters on the same bars.

In addition to the ASC/GSH cycle that lies at the core of the plant’s ability to metabolize ROS, direct ROS-detoxifying enzymes (SOD, CAT, POX, GR, and GPX) are well known in reducing ROS accumulation in stressed plants. When As^III^ compared to non-stressed plants, SOD, CAT, POX, GR, and GPX activity significantly increased in the wheat plant leaves (Figure 6). However, the activities of SOD, CAT, POX, ASC, GR, and GPX enzymes were increased significantly due to the treatments (i.e., OSW, AMF, and combination (OSW+AMF)) compared to As^III^-stressed plants. The combination (OSW+AMF) mitigated the adverse effects of As^III^ stress, where the SOD activity was increased by 97%, CAT raised by 121%, POX activity was increased by 105%, GR activity was raised by 129%, and GPX activity was raised by 110% compared to As^III^ stress (Figure 6).

### 2.7. Anthocyanin Metabolism in Wheat Plants under As^III^ Stress

**Figure 7:**
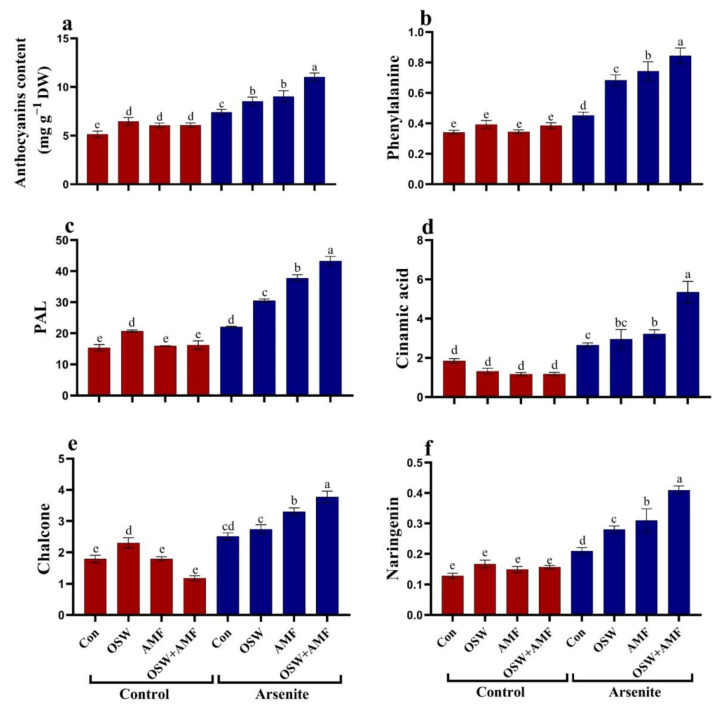
The influence of olive solid waste (OSW) and arbuscular mycorrhizal fungi (AMF), combination (OSW + AMF) on anthocyanin metabolism (Anthocyanin (**a**), Phenylalanine (**b**), Phenylalanine aminolyase (PAL) (**c**), Cinnamic acid (**d**), Chalcone (**e**), and Naringenin (**f**) in wheat plants growing under As stress. Fishers test at *p* < 0.05 reveals significant variations in means (±standard deviation), which are different letters on the same bars.

In As^III^-stressed plants, anthocyanin level and metabolism (PAL enzyme activity and cinnamic acid, chalcone, and naringenin levels) were increased significantly compared to the non-stressed plants (Figure 7). Stressed wheat plants with 100 mg/kg As^III^ show significant increases in anthocyanin level. These increases prompted us to evaluate the changes in biosynthesis intermediates of anthocyanins (phenylalanine, cinnamic acid, and naringenin content) as well as key biosynthetic enzymes (PAL and CHS) (Figure 7). In this regard, anthocyanin, phenylalanine, PAL, cinnamic acid, and naringenin content were increased by approximately 44%, 32%, 43%, 44%, 40%, and 63% respectively, compared to untreated plants (Figure 7). The combination (OSW+AMF) mitigated the adverse effects of As^III^ stress, where the Anthocyanin levels were increased by 48%, phenylalanine by 86%, PAL activity was raised by 95%, cinnamic acid content increased by 101%, chalcone raised by 50%, and naringenin raised by 95% compared to As^III^ stress (Figure 7).

## 3. Discussion

In the present study, As^III^ contamination at an applied level (100 mg/Kg soil) negatively affected wheat growth and yield by limiting nutrient uptake and overall plant development. This agrees with previous findings [6]. Arsenic is not an essential nutrient but could be taken up by the plant roots and translocated to different parts of the plant [25,26]. As noted by Wu et al., [6], As is a chemical analogue of P; hence, it is taken up by plant roots via P transporters. By its accumulation in plant tissues, this hazardous element has the potential to enter the food chain and cause harmful effects to human and animal health [27]. Its availability in the soil inhibits plant growth development by limiting root growth and nutrient uptake [28]. This is achieved through As-induced inhibition of root extension, proliferation, and interference with critical metabolic and cellular processes [29]. Therefore, it is important to reduce As toxicity in wheat plants [30].

In the current study, wheat plants were cultivated in As^III^-contaminated soil without or with OSW and/or AMF at plant growth. These results are consistent with earlier studies that found As^III^ toxicity in a variety of plant species [31]. The deleterious effects of As^III^ on plant growth, mineral accumulation, and physiology might explain this decrease in wheat development [32]. Our results demonstrated that AMF inoculation positively influenced plant growth traits. A combination of OSW + AMF has various effects on plant growth under harsh conditions [33]. Changing the metal species in the soil, a combination of OSW+AMF may minimize metal build-up in plants, resulting in increased plant growth and biomass. Under experimental conditions, adding a combination of OSW + AMF boosted the development of As^III^-stressed wheat. In this research, OSW and/or AMF enhanced shoots’ fresh and dry weights in wheat plants under both control and under As^III^ stress. However, at 100 mg/kg, the As^III^ form proved poisonous to the wheat plants, causing a significant growth decline. Vejvodová et al., [15] showed biomass losses in wheat under As stress, as well as those that showed OSW and/or AMF alleviating As^III^-toxic effects on plant growth. Furthermore, As^III^ negatively influenced AMF colonization by lowering root colonization levels, suggesting fungal toxicity.

To further understand the impacts of As^III^ stress on the plant-OSW and/or AMF systems, we used an approach that considers how soil metal concentrations might influence plant metal concentrations either directly via root absorption or indirectly through effects on AMF colonization (Table 1). AMF colonization was evidently considerably decreased in both examined plants in response to As^III^ toxicity. AMF may resist metal stress by reducing the intake of non-essential metals or by sequestering metals in cell walls or intracellular compartments to elicit cell damage [34]. AMF may also influence metal mobility and availability in soil by generating exudates that chelate or bind metals [35]. AMF, for example, may influence plant metal intake by either decreasing plant uptake or sequestering metals in their own tissues or enhancing plant uptake by actively translocating metals [34]. Depending on the colonization state of the plants, AMF may either ease or worsen the toxicity of As^III^ stress in plants. The link between AMF colonization and plant metal absorption, on the other hand, is not always linear [36]. Consequently, synthesizing these divergent findings across multiple plant–metal systems is challenging, and most do not explore the potential that soil metals influence plants directly and indirectly via AMF [34]. 

Arsenite triggers the production of ROS, which have detrimental impacts on plants at biochemical, physiological, and molecular levels. The role of different enzymatic (SOD, CAT, GR, and APX) and non-enzymatic (salicylic acid (SA), proline, phytochelatins, GSH, nitric oxide, and phosphorous) substances under As stress has been defined via conceptual models showing As^III^ translocation and toxicity pathways in plants [12]. Several biochemical compounds such as chlorophyll, CAT, and proline were found to be significantly changed in mung bean crops under As^III^ stress [37]. The changes in these compounds negatively impact the growth and development of mung bean crops and other food crops. Among these parameters, increases in the chlorophyll enhance photosynthesis and increase the green color in food crops.

OSW, AMF, and combination OSW+ AMF have all shown that our As^III^ research significantly improves all photosynthetic criteria. Zhang et al. [38] results are corroborated by OSW and/or AMF, which reveal that an increase in biosynthetic pathway intermediates and metabolites, necessary for chlorophyll synthesis under As stress is the sole component leading to an increase in photosynthetic rate and efficiency. Furthermore, enhanced photosynthetic rate, sugar build-up, and reduced oxidative stress have all been employed to illustrate OSW’s efficacy in heavy metal stress conditions [39]. The amount of As^III^ pollution in the soil and pigment concentrations were considerably higher in OSW- and/or AMF-treated plant shoots. Chl a and Chl b concentrations in OSW and/or AMF plants have been observed to be higher in citrus, cucumber, and chickpea plants [39].

When exposed to As^III^ stress, a greater concentration of total carotenoids (OSW + AMF) than AMF and OSW imply that Chl is protected from As^III^-induced oxidative damage. Extensive reactive oxygen species (ROS) occur during As^III^ stress, just as they do under other stressful conditions. Arsenite a result, large levels of oxidative biomarkers such as MDA and H_2_O_2_ were dramatically elevated in response to environmental challenges and employed as a positive criterion to assess plant resistance to As^III^ stress. This research found that all oxidative indicators were reasonably low by using OSW, AMF, and a mixture of the levels seen in As^III^-stressed plants. Physiologically, the higher frequency of lipid peroxidation and H_2_O_2_ concentration in wheat plants has been linked to enhanced plasma membrane breakdown and cytoplasm dryness caused by severe As^III^ stress. Furthermore, H_2_O_2_ is responsible for the enhanced solute breakdown associated with oxidative stress [40].

OSW and/or AMF have the ability to reduce oxidative stress by preserving plasma membrane integrity, regulating water intake, and enhancing water consumption efficiency [41]. Similar to the positive effect of OSW, is the possible influence of biochar on crop yield and biomass production in bean plants under As^III^ stress conditions on MDA and H_2_O_2_ levels [42]. According to Sharma et al. [35], AMF was also indicated to reduce MDA to a minimum level in order to counteract As^III^ stress. Redox equilibrium and the antioxidant defense system are critical in controlling the formation of excessive ROS and the duration of oxidative stress in plants under irregular environmental circumstances [43]. OSW and/or AMF have been identified as a pleiotropic component that directly or indirectly assists in detoxifying excess ROS by reinforcing the antioxidant defense system, resulting in enhanced stress tolerance [43]. In wheat plants treated with OSW and/or AMF in the presence of As^III^ stress, the ascorbate-glutathione cycle enzymes (APX, GR, GST, DHAR, and MDHAR) were also upregulated, which may serve to scavenge high H_2_O_2_ by balancing ASC and GSH levels. In addition, the ASC-GSH cycle enzymes’ effective actions help to maintain cellular redox equilibrium and prevent oxidative damage [43]. Under high stress, the ASC-GSH pool in cucumber was similarly disrupted [43].

In addition to antioxidant enzymes, polyphenols mitigated As^III^ toxicity by trapping the hydroxyl radical [13,14,15,16,17,18,19,20,21,22,23,24,25,26,27,28,29,30,31,32,33,34,35,36,37,38,39,40,41,42,43]. The flavonoid also acts as an antioxidant and antiradical by chelating transition metals (Fe^2+^), preventing the Fenton reaction from creating ROS. Consistently, co-cultivation with OSW and/or AMF, as well as As^III^ treatment, boosted the concentration of phenols and flavonoids even more. This increase may be attributed to the activation of the phenylpropanoid pathway through increased PAL activity. We also assessed PAL, the rate-limiting biosynthetic enzyme for phenols and flavonoids, which was enhanced in As^III^-stressed plants in the presence or absence of OSW and/or AMF treatment, supporting the hypothesis that greater PAL content may aid in boosting phenols and flavonoids contents [44]. In line with our results, CHS transcript levels increased in As^III^-stressed plants, and the expression increased even more following melatonin treatment, indicating that CHS may assist in improving anthocyanin concentration and minimizing toxicity [43]. Our findings are also supported by studies of similar melatonin properties in pepper plants exposed to boron poisoning and rosemary herb exposed to arsenic [45]. Furthermore, anthocyanins may behave as the Swiss Army knife of the plant world, defending plants against stress in a number of ways [46]. Similarly, OSW and/or AMF significantly enhanced anthocyanin levels in plants sprouts, demonstrating higher antioxidant activities, reduced ROS formation, and osmotic adjustment indicators (OSW and/or AMF significantly enhanced anthocyanin levels in plants sprouts, demonstrating higher antioxidant activities, reduced ROS formation, and osmotic adjustment indicators [47]. In reaction to stress, anthocyanins quench H_2_O_2_ to preserve cell homeostasis. However, the activity of the chloroplastic antioxidant enzymes is decreased at this moment [48]. Notably, anthocyanins have been shown to behave as superoxide anions and H_2_O_2_ scavengers in the vacuoles of mesophyll cells [49]. The enzyme chalcone synthase is actively involved in producing anthocyanins [49].

## 4. Material and Methods

### 4.1. Experimental Set up

In the experiment, 30% soil (300 g) and sterilized 70% sand (700 g), each with a water content of 60%, were mixed with Tref EGO substrates (50 g) in Moerdijk, NL. In pot experiment, soils were supplemented with 4% *w/w* of solid olive waste and placed into 2 L pots. Olive solid waste was obtained from the three-phase olive oil factory in Al Juef, Saudi Arabia. This dose was selected according to a preliminary experiment testing different concentrations of solid olive waste (0–10%, *w/w*) on wheat growth. Treated and untreated soil containing solid olive waste was separated into two groups: inoculated with *Rhizophagus irregularis* (AMF; MUCL 41833). Surface sterilization of the seeds was accomplished using sodium hypochlorite (5% *v/v*, 20 min). Wheat seeds (*Triticum aestivum* L., cv Giza 119) were received from the Giza Agriculture Research Center in Egypt. At a humidity of 0.33 g water/g dry soil, the soil originally contained 14.8 mg nitrate-nitrogen (N), 10.7 mg carbon (C), 1.1 mg ammonium-N, and 9.4 mg phosphorus (P)/g air-dry soil. The pH was 7.56, the 3.4 dS/m EC, and the K concentration (2.75 meq/L). Seeds were germinated in T1) control soil, T2) soil treated with olive solid waste (OSW, 4% *w/w*), T3) AMF-inoculated soil, T4) AMF and OSW treated soil, T5) As^III^ treated soil (as NaAsO_2_ at 100 mg/kg soil), T6) soil treated with AS^III^ and OSW, T7) soil treated with AS^III^ + AMF, and T8) soil treated with As^III^, OSW and AMF (Table 2).

Throughout the experiment, the soil water content was held at 60% (maximum water holding capacity). Based on early studies, a concentration of 100 mg of As^III^ per kg of soil was chosen to excrete stress, which caused a 50% decrease in plant development. Because As^III^ is more poisonous, mobile, and soluble than other As forms, like As^V^. Plants were cultivated in a climate-controlled room at 21/18 °C with a day/night photoperiod of 16/8 h (60% humidity, 150 mol PAR m2 s1). All pots were set up in a randomized full-block design with five replicates for each treatment. Tap water was used to water the pots daily. The rhizosphere soils and plant shoots and roots were collected after 6 weeks of seed sowing. Plant shoot tissues were collected in liquid N and kept at −80 °C for subsequent biochemical analysis. Soil samples were collected in an icebox and kept at −20 °C for subsequent physical and biological examination. The fresh and dried weights of the harvested shoots and roots were calculated, and AMF related parameters (root colonization, hyphal length, and number of arbuscules) were analyzed.

Fresh roots were rinsed with tap water, split into segments (0.5–1 cm), and cleaned with a 10% KOH solution for each treatment. The roots were then stained with 0.05% trypan blue (SIGMA) in lacto-glycerol and inspected under a stereomicroscope. The colonization was then computed using the gridline intersect technique, and the abundance of arbuscular in the root system was determined by the number of arbuscules cm^−1^ root [50]. Finally, the hyphal length was calculated using the methods of Tennant, [51] and Rillig et al., [52].

### 4.2. Soil Analysis

By shaking the excavated roots gently, the rhizosphere was separated from the bulk soil, and then soil attached to the fine roots (0–2-mm thick layer) was brushed [38]. described that phenolic content was spectrophotometrically determined for 10 g of the rhizosphere in distilled water using a Shimadzu UV–Vis 1601 PC. In addition, according to [53], mineral nutrients and organic matter (OM) were measured (Table 3). Finally, according to Brown et al. [54], CaCO_3_ percentages were estimated. This study used CN element analyzers (NC-2100, Carlo Erba Instruments, Milan, Italy) to determine the C and N contents. Using ribitol as an internal standard, citric acid was extracted in a 0.1% phosphoric acid solution containing butylated hydroxyanisole. De Sousa et al. [55] describe HPLC quantification of the centrifuged filtrates (LaChrom L-7455 diode array, LaChrom, Tokyo, Japan). Nitric acid (HNO_3_) and hydrochloric acid (HCLO_4_) were used to digest soil and plant samples to extract total As. After stranding the samples overnight, they were transported to the digestion block at 120 °C and halted when a thick white fume of HCLO_4_ was released. By using external calibration, flow injection hydride generation atomic absorption spectrophotometry (FI-HG-AAS, Perkin Elmer A Analyst 400, CITY, USA) was used to analyze the As concentration in plant tissues and soil after digestion [56]. Maximum sensitivity was obtained using 10% HCl and 0.4% NaBH_4_.

### 4.3. Photosynthesis Related Parameters

Based on data from AbdElgawad et al. [57], we determined the light-saturated photosynthetic rate and gas exchange of developed leaves utilizing the COR LI-6400, LI-COR Inc., Lincoln, NE, USA. To measure pigment concentration, 80% acetone was used to extract pigments [57].

### 4.4. Stress Markers

50 mg tissues (leaves and properly rinsed root discs) were extracted in 1 mL 80% ethanol using MagNALyser to determine lipid peroxidation levels (Roche, Vilvoorde, Belgium). The extract was then tested for malondialdehyde (MDA) using the thiobarbituric acid assay [58]. Protein carbonyls were evaluated as oxidative damage indicators using Cayman Chemical’s (Ann Arbor, MI). Protein Carbonyl Colorimetric Assay Kit [59]. The Xylenol orange technique, which relies on peroxide-catalyzed oxidation of Fe^2+,^ was used to measure hydrogen peroxide (H_2_O_2_) in trichloroacetic acid (TCA) (0.1%) extracts of plant materials [60]. To rule out non-specific interactions, each sample was matched to its catalase-treated counterpart.

### 4.5. Antioxidant Metabolites

Total antioxidant capacity (ferric reducing antioxidant power, FRAP) was extracted in ice-cold 80% ethanol using a MagNALyser (Roche, Vilvoorde, Belgium) and measured using Trolox as a reference [55]. Plant materials were extracted in 1mL 80% ethanol using MagNALyser for ascorbate and glutathione measurement (Roche, Vilvoorde, Belgium). HPLC was used to measure reduced ascorbate (ASC) and reduced glutathione (GSH). Total ascorbate (ASC + DHA) and glutathione (GSH + GSSG) concentrations were evaluated after dithiothreitol (DTT) reduction, as previously reported [13]. Polyphenols and flavonoids were extracted by homogenizing fresh plant materials in 80% ethanol and centrifuged for 15 min at 5000 rpm. The clear extract was then used to determine the total phenolic and flavonoid content using the Folin-Ciocalteu and aluminum chloride tests.

### 4.6. Antioxidant Enzyme Activities

A MagNALyser (Roche, Vilvoorde, Belgium) was used to homogenize 100 mg of frozen plant materials in 1 mL buffer [50 mM potassium phosphate, pH 7.0, 10% (*w/v*) polyvinyl pyrrolidone (PVP), 0.25% (*v/v*) Triton X-100, 1 mM phenylmethylsulfonyl fluoride (PMSF), 1 mM ASC]. After 10-min centrifugation at 13,000 rpm and 4 °C, the clear supernatant was used to assess the activities of several antioxidant enzymes such as superoxide dismutase (SOD, EC (Enzyme Commission number) 1.15.1.1), peroxidase (POX, EC 1.11.1), catalase (CAT, EC1.11.1.6), glutathione peroxidase (GPX, EC 1.11.1.9), and ascorbate peroxidase (APX (GST, EC 2.5.1.18). Dhindsa et al. [61] evaluated SOD activity by measuring the inhibition of nitroblue tetrazolium (NBT) reduction at 560 nm. POX activity was evaluated using the technique developed by Kumar and Khan, [62] based on pyrogallol oxidation (ε 430 = 2.47 mM^−1^.cm^−1^). The breakdown of H_2_O_2_ at 240 nm (ε 240 = 0.0436 mM^−1^.cm^−1^) was used to assess CAT activity [55]. Murshed et al. [63] methods were used to measure the activity of APX, monodehydroascorbate reductase (MDHAR), dehydro-ascorbate reductase (DHAR), and GR enzymes, by using microplate assay methods. Drotar et al. [64] measured GPX activity as a reduction in NADPH absorbance at 340 nm (ε 340 = 6.22 mM^−1^.cm^−1^)). 

### 4.7. Statistical Analysis

A completely randomized block design was used in the experiments.

The experiments were conducted using a completely randomized block design. SPSS (Chicago, IL, USA) and R were used to analyze the data (Team, 2013). The Kolmogorov-Smirnov (SPSS) and Levene’s tests were used to determine data normality and homoscedasticity. Duncan’s (SPSS, following two-way ANOVA) was used for the subsequent pairwise statistical comparison of means.

## 5. Conclusions

This study aimed to understand how OSW can stimulate the AMF role in As^III^ stress mitigation in wheat plants. Increased AMF colonization under OSW treatment improved growth and physiology of wheat plants, particularly under As^III^ stress. AMF and/or OSW also provides C skeletons for production antioxidants, which might be one of the main reasons for improving plant tolerance to As^III^ toxicity. To understand further the signaling and molecular mechanisms of OSW-induced AMF positive impact under As^III^ stress, underlying detailed metabolomics and transcriptomic studies are needed.

## Figures and Tables

**Table 1:** Root colonization, hyphal length, and No. of arbuscules of wheat plants in the different treatments (AMF, and/or As^III^ treatments). Three biological replicates per treatment are analyzed. Values are presented as mean ± SE. Means sharing the same letters, for a parameter within a species, do not differ significantly at *p* ≤ 0.05.

	Root Colonization (%)	Hyphal Length (cm g^−1^ Soil)	Arbuscules Numbers (cm^−1^ Root)
AMF	56.98 ± 6.45 a	24.17 ± 3.76 b	6.04 ± 0.37 a
AMF+OSW	61.8 ± 2.3 a	26.17 ± 3.0 a	5.95 ± 0.4 a
As^III^ + AMF	30.8 ± 1.18 c	12.26 ± 0.48 c	3.6 ± 0.68 c
As^III^ + OSW + AMF	34.01 ± 7.39 b	13.63 ± 1.46 c	4.2 ± 0.23 b

**Table 2:** A list of applied treatments.

Treatment Code	Treatments
T1	Control soil
T2	Soil treated with olive solid waste (OSW, 4% *w/w*)
T3	AMF-inoculated soil
T4	AMF and OSW treated soil
T5	As^III^-treated soil (as NaAsO_2_ at 100 mg/kg soil)
T6	Soil treated with As^III^ and OSW
T7	Soil treated with As^III^ + AMF
T8	Soil treated with As^III^, OSW and AMF

**Table 3:** Physico-chemical characterization of olive solid waste (OSW). Three sampels of OSW were analyzed. Values are presented as mean ± SE.

Physico-Chemical Characterization	
Water content (%)	85.46 ± 5.93
Dry matter (%)	13.36 ± 1.47
pH	5.96 ± 0.42
Electric conductivity (mS cm^−1^ )	14.46 ± 1.03
Chemical oxygen demand (g L^−1^ )	101.49 ± 11.2
Biochemical oxygen demand (gL^−1^)	52.11 ± 5.75
Organic matter (g L^−1^)	64.65 ± 4.6
Salinity (g L^−1^ )	7.21 ± 0.51
*Minerals*	
Nitrogen (N) (g L^−1^)	1.96 ± 0.14
Phosphorus (P) (g L^−1^)	1.72 ± 0.19
Potassium (K) (g L^−1^)	9.71 ± 1.07
Calcium (Ca) (g L^−1^)	0.93 ± 0.07
Magnesium (Mg) (g L^−1^)	0.5 ± 0.04
Sodium (Na) (g L^−1^)	1.78 ± 0.13
Chloride (Cl) (g L^−1^)	1.25 ± 0.09
Iron (Fe) (g L^−1^)	0.71 ± 0.05
Zinc (Zn) (g L^−1^)	0.18 ± 0.01
*Antioxidant*	
Antioxidant Activity (FRAP)	53.66 ± 4.98
Antioxidant Activity DPPH (%)	65.34 ± 9.71
*Phenols*	
Total phenols (g L^−1^)	3.96 ± 0.97
Flavonoids (g L^−1^)	1 ± 0.24
Caffeic acid	0.03 ± 0
Ferulic acid	3.23 ± 0.16
Protocatechuic acid	0.32 ± 0.03
Catechin	0.97 ± 0.05
Galic acid	20.88 ± 2.48
p-Coumaric acid	4.57 ± 0.5
Resorcinol	0.04 ± 0.01
Chlorogenic acid	0.26 ± 0.04
Syringic acid	1.43 ± 0.08
Quercetin	0.06 ± 0
Quercetrin	0.1 ± 0.01
Luteolin	0.07 ± 0.03
Apigenin	0.4 ± 0.24
Isoquercetrin	0.45 ± 0.04
Rutin	0.05 ± 0
Ellagic acid	0.02 ± 0
Velutin	0.02 ± 0
Naringenin	0.07 ± 0.01
Genistein	0.11 ± 0.01
Daidzein	0.16 ± 0.01
Fisetin	0.21 ± 0.02
O-hydroxydaidzein	0.02 ± 0.01

## Data Availability

The data presented in this study are available upon request from the corresponding author.
